# Preferential Th1 Cytokine Profile of Phosphoantigen-Stimulated Human V**γ**9V**δ**2 T Cells

**DOI:** 10.1155/2010/704941

**Published:** 2011-02-21

**Authors:** Margaret R. Dunne, Bozgana A. Mangan, Laura Madrigal-Estebas, Derek G. Doherty

**Affiliations:** ^1^Department of Immunology and Institute of Molecular Medicine, Trinity College Dublin, St. James's Hospital, Dublin 8, Ireland; ^2^Institute of Immunology, National University of Ireland, Maynooth, Co. Kildare, Ireland; ^3^National Children's Research Centre, Our Lady's Hospital for Sick Children, Crumlin, Dublin 12, Ireland

## Abstract

Human V**γ**9V**δ**2 T cells recognise pyrophosphate-based antigens (phosphoantigens) and have multiple functions in innate and adaptive immunity, including a unique ability to activate other cells of the immune system. We used flow cytometry and ELISA to define the early cytokine profiles of V**γ**9V**δ**2 T cells stimulated in vitro with isopentenyl pyrophosphate (IPP) and (E)-4-hydroxy-3-methyl-but-2 enyl pyrophosphate (HMB-PP) in the absence and presence of IL-2 and IL-15. We show that fresh V**γ**9V**δ**2 T cells produce interferon-**γ** (IFN-**γ**) and tumour necrosis factor-**α** (TNF-**α**) within 4 hours of stimulation with phosphoantigen, but neither IL-10, IL-13, nor IL-17 was detectable up to 72 hours under these conditions. Cytokine production was not influenced by expression or lack, thereof, of CD4 or CD8. Addition of IL-2 or IL-15 caused expansion of IFN-**γ**-producing V**γ**9V**δ**2 T cells, but did not enhance IFN-**γ** secretion after 24–72 hours. Thus, phosphoantigen-stimulated V**γ**9V**δ**2 T cells have potential as Th1-biasing adjuvants for immunotherapy.

## 1. Introduction

*γδ* T cells constitute approximately 1–5% of circulating human T lymphocytes, and the majority (typically >80%) of these express T-cell receptors (TCRs) consisting of V*γ*9 and V*δ*2 chains. The V*γ*2V*δ*2 TCR recognises small, nonpeptide phosphate antigens (phosphoantigens) independently of major histocompatibility complex (MHC) molecules (reviewed in references [1, 2]). This *γδ* T-cell subset exists only in humans and higher primates, where numerous roles in immunosurveillance and protection against infection and tumours have been hypothesised, yet a specific, unique role remains to be found. *In vitro* studies have shown that activated V*γ*9V*δ*2 T cells rapidly produce an array of proinflammatory cytokines and chemokines [3], cytolytic compounds [4, 5], antiviral [6], antimicrobial [7], and tissue growth factors [8, 9]. Activating crosstalk has been described between V*γ*9V*δ*2 T cells and other immune cells, such as neutrophils [10], B cells [11], monocytes [12], and dendritic cells [13–16]. The V*γ*9V*δ*2 T-cell subset has also been shown to have antigen presentation and cross-presentation capabilities comparable to those of professional antigen presenting cells [17, 18]. The basis for this apparent functional pleiotropy is unknown but is likely to be influenced by the conditions under which *γδ* T cells become activated, such as in the absence or presence of an ongoing inflammatory response. 

The imperative to reliably define V*γ*9V*δ*2 T cell responses is now more urgent, since these cells and their activating antigens are currently of interest as immunotherapeutic agents, particularly for treatment of cancer. In addition to their antigen presenting [17, 18] and adjuvant [11–16] activities, V*γ*9V*δ*2 T cells recognise and kill a range of tumour cells *in vitro* [5, 19–21], and clinical trials in cancer patients are ongoing [22–26]. Current treatment strategies aim to optimally expand and activate V*γ*9V*δ*2 T cells (either ex vivo or *in vivo*) by administering phosphoantigens along with T-cell growth factors, most commonly IL-2, or by using aminobisphosphonates which inhibit isoprenoid synthesis resulting in the accumulation of endogenous phosphoantigens [27]. Such strategies have shown evidence of improved patient survival; however, the observed plasticity of V*γ*9V*δ*2 T cell functions presents a double-edged sword, with the potential to treat disease balanced against the possibility of triggering undesirable immune responses, or cell exhaustion. Thus, further characterisation of activated V*γ*9V*δ*2 T cells is required if such cells are to be used to generate reliable immunological outcomes.

Numerous studies have demonstrated that murine *γδ* T-cell subsets can produce cytokines that control distinct adaptive immune responses, such as those categorised as T helper type 1 (Th1), Th2, Th17, or regulatory T (Treg) responses [28–32]. Human V*γ*9V*δ*2 T cells can readily release the Th1 cytokines IFN-*γ* and TNF-*α* [12, 14, 33–37], and subsets of these cells can be induced under certain conditions to produce Th2 (IL-4, IL-5 and IL-13), Th17 (IL-17), and Treg (IL-10) cytokines [11, 35, 38, 39]. In the present study, we have characterised the early Th cytokine profiles of primary V*γ*9V*δ*2 T cells taken from the peripheral blood of healthy volunteers and stimulated *in vitro* with phosphoantigens in the presence and absence of T-cell growth factors IL-2 or IL-15. The antigens used were isopentenyl pyrophosphate (IPP) and (E)-4-hydroxy-3-methyl-but-2 enyl pyrophosphate (HMB-PP), intermediates of the mevalonate and nonmevalonate pathways of isoprenoid synthesis, respectively [40, 41]. HMB-PP is the most potent *γδ* T cell stimulating antigen described to date [40] but has yet to be clinically exploited. Our results show that V*γ*9V*δ*2 T cells stimulated with this novel phosphoantigen under clinically relevant conditions have similar cytokine profiles to IPP-stimulated V*γ*9V*δ*2 T cells, with rapid production of cytokines that promote Th1 responses, but little or no cytokines associated with Th2, Th17, or Treg responses.

## 2. Materials and Methods

### 2.1. Blood Samples and Preparation

Anticoagulated venous blood samples were obtained from healthy donors. For *γδ* T-cell enrichments, blood from buffy coat packs from the Irish Blood Transfusion Service Board was used. Peripheral blood mononuclear cells (PBMCs) were prepared by standard density gradient centrifugation over Lymphoprep (Nycomed, Oslo, Norway).

### 2.2. Magnetic Bead Enrichment of *αβ* and *γδ* T Cells

*γδ* T cells were enriched from PBMC by positive selection using the Anti-TCR *γδ* Microbead Kit (Miltenyi Biotec, Bergisch Gladbach, Germany). T cells negative for the *γδ*TCR (hereafter denoted *αβ* T cells) were prepared by positive selection of CD3^+^ cells from the *γδ* TCR-negative fractions using CD3 Microbeads (Miltenyi Biotec). Purity of *αβ* and *γδ* T-cell fractions was assessed by flow cytometry.

### 2.3. In Vitro Stimulation of T Cells

PBMC or enriched *αβ* and *γδ* T cells were plated at 10^6^ cells/mL in complete RPMI medium (RPMI 1640 containing Glutamax supplemented with 25 mM HEPES, 50 mg/mL streptomycin, 50 U/mL penicillin, 2 *μ*g/mL Fungizone, and 10% heat-inactivated foetal calf serum) and stimulated for 4–72 hours with phorbol myristate acetate (PMA; 10 ng/mL; Sigma-Aldrich, Poole, UK) and ionomycin (1 *μ*g/mL; Sigma-Aldrich) or various concentrations of the phosphoantigens IPP (0–100 *μ*M; Sigma-Aldrich) or HMB-PP (0-1 *μ*M; kindly donated by Hassan Jomaa and Armin Reichenberg) [42], with and without IL-2 (50 U/mL; donated by National Cancer Institute—Frederick Research Foundation Biological Resources Branch) or IL-15 (10 ng/mL; R&D Systems, Abingdon, UK).

### 2.4. Intracellular Analysis of Cytokine Production by V*γ*9V*δ*2 T Cells

Total PBMCs were stimulated for 4 hours as described in Section 2.3 in the presence of brefeldin A (10 *μ*g/mL, Sigma-Aldrich) to promote intracellular accumulation of cytokines. Cells were harvested and stained for cell surface expression of V*γ*9, CD3, CD4, and/or CD8 and intracellular expression of IFN-*γ*, TNF-*α*, IL-10, IL-13, or IL-17 using fluorochrome-conjugated monoclonal antibodies (mAb) obtained from BD Biosciences, (Oxford, UK), Immunotools (Friesoythe, Germany), eBioscience (Hatfield, UK), and R&D Systems (Abingdon, UK) [43]. Cells were analysed using a FACSCalibur flow cytometer with CellQuest software (BD Biosciences) or with a CyAN-ADP flow cytometer with Summit (Beckman Coulter) and FloJo (Ashland, OR) software. Unstimulated control samples were incubated alongside test samples and isotype-matched, nonspecific mAbs, and fluorescence-minus-one controls were used to set compensations and gates.

### 2.5. Cytokine Quantification by ELISA

*αβ* and *γδ* T cells were enriched from PBMC by magnetic bead selection as described in Section 2.2 and 0.2 × 10^6^ cells per well of a 96-well plate were stimulated *in vitro* as described in Section 2.3. Supernatants from these cultures were harvested 24, 48, and 72 hours after stimulation and the levels of IFN-*γ*, IL-10, IL-13, and IL-17 were quantified by ELISA using antibody pairs purchased from R&D Systems (DuoSet ELISA Development kits).

### 2.6. Statistical Analyses

Statistical analysis of data was carried out using Prism GraphPad Version 5.0. Differences between groups were assessed using paired *t*-test, and *P* values of <.05 were considered significant.

## 3. Results

### 3.1. V*γ*9V*δ*2 T Cells Rapidly Produce IFN-*γ*, But Not IL-10, IL-13, Nor IL-17, in Response to Pyrophosphate Stimulation

**Figure 1:**
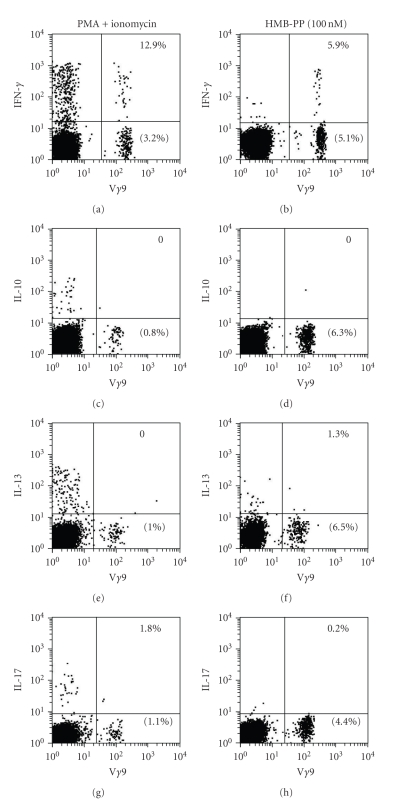
V*γ*9V*δ*2 T cells rapidly produce IFN-*γ*, but not IL-10, IL-13, nor IL-17, in response to PMA and ionomycin or pyrophosphate stimulation. (a) Flow cytometry dot plots showing IFN-*γ*, IL-10, IL-13, and IL-17 expression by V*γ*9^+^ and V*γ*9^−^ PBMC after 4-hour stimulation with PMA and ionomycin or HMB-PP, in the presence of brefeldin A. Plots are representative of experiments on PBMC from 8 donors. Numbers in the lower right hand quadrants indicate the percentages of lymphocytes that express the V*γ*9 TCR. Numbers in the upper right hand quadrants indicate the percentages of V*γ*9^+^ T cells that produce each cytokine.

Early cytokine production by V*γ*9V*δ*2 T cells was examined by stimulating freshly isolated PBMC for 4 hours with medium alone, PMA and ionomycin, or various concentrations of IPP or HMB-PP in the presence of brefeldin A (10 *μ*g/mL). Cells were then washed and stained for cell surface expression of CD3 and V*γ*9 or V*δ*2 and intracellular expression of IFN-*γ*, IL-10, IL-13, or IL-17. Virtually all V*γ*9^+^ cells expressed the V*δ*2 chain and vice versa, with single-positive V*γ*9^+^ or V*δ*2^+^ being very rare [16]; therefore, V*γ*9V*δ*2 T cells were subsequently identified by a single V*γ*9 mAb stain. We found that the numbers of V*γ*9^+^ cells detectable by flow cytometry after stimulation of PBMC with PMA and ionomycin were usually lower than those detectable in unstimulated PBMC from the same donors (not shown) or after stimulation with phosphoantigen (Figure 1). This may be due to proliferation of other non-V*γ*9^+^ cells or downregulation of TCR expression by V*γ*9V*δ*2 T cells, which occurs after stimulation with PMA and ionomycin but not HMB-PP [44].

Analysis of early cytokine production by fresh PBMC from 8 healthy donors showed that V*γ*9 cells produced IFN-*γ*, but not IL-10, IL-13, nor IL-17 within 4 hours of stimulation with either IPP (data not shown), HMB-PP (Figure 1), or PMA and ionomycin (Figure 1). The percentages of V*γ*9^+^ cells that produced IFN-*γ* did not increase with greater stimulation times (data not shown). Cytokine production by non-V*γ*9V*δ*2 cells never exceeded background levels (0.4%) in response to phosphoantigens yet was evident in response to PMA and ionomycin treatment, for all cytokines assayed (Figure 1).

### 3.2. IL-2 and IL-15 Augment IFN-*γ* Expression But Do Not Induce IL-10, IL-13, Nor IL-17 Expression by Phosphoantigen-Stimulated V*γ*9V*δ*2 T Cells

**Figure 2:**
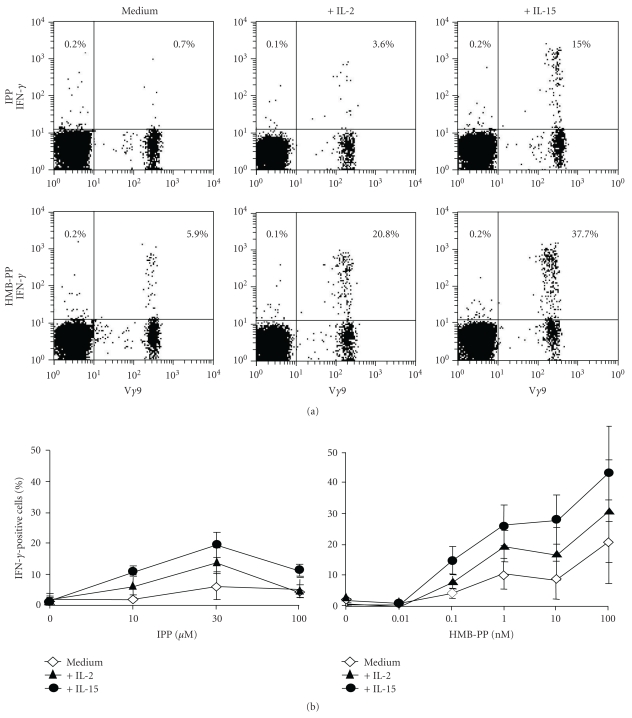
IL-2 and IL-15 augment IFN-*γ* expression by phosphoantigen-stimulated V*γ*9V*δ*2 T cells. (a) Representative dot plots showing IFN-*γ* expression by V*γ*9 cells 4 hours after stimulation with IPP (10 *μ*M) or HMB-PP (10 nM), in the absence or presence of 50 U/mL IL-2 or 10 ng/mL IL-15. Plots are representative of 8 independent studies. Numbers in the plots indicate the percentages of V*γ*9^−^ (left) and V*γ*9^+^ (right) T cells that produce each cytokine. (b) Mean (± SEM) percentages (*n* = 8) of V*γ*9 cells expressing IFN-*γ* after stimulation with various concentrations of IPP or HMB-PP, in the absence and presence of IL-2 and IL-15.

The frequencies of V*γ*9V*δ*2 T cells that produced IFN-*γ* in response to stimulation of PBMC with IPP or HMB-PP were enhanced by adding IL-2 or IL-15 to culture media (Figure 2(a)). Addition of these growth factors did not lead to the induction of IL-10, IL-13, nor IL-17 production by phosphoantigen-stimulated V*γ*9V*δ*2^+^ or V*γ*9V*δ*2^−^ T cells (not shown). The percentages of V*γ*9V*δ*2 T cells producing IFN-*γ* increased in a dose-dependent manner in response to IPP and HMB-PP, with half-maximal stimulation occurring when 10 *μ*M IPP or 1 nM HMB-PP were used (Figure 2(b)). Therefore, as previously reported [40, 41], HMB-PP was ~10,000-fold more potent than IPP at stimulating IFN-*γ* production by V*γ*9V*δ*2 cells. While the presence of IL-2 or IL-15 in cell stimulations led to increases in the percentages of V*γ*9V*δ*2 T cells that produced IFN-*γ*, it did not significantly lower the concentrations of phosphoantigens that were required to induce IFN-*γ* expression (Figure 2(b)).

### 3.3. *γδ* T Cells Release Maximal Amounts of IFN-*γ* 72 Hours after Phosphoantigen Stimulation

**Figure 3:**
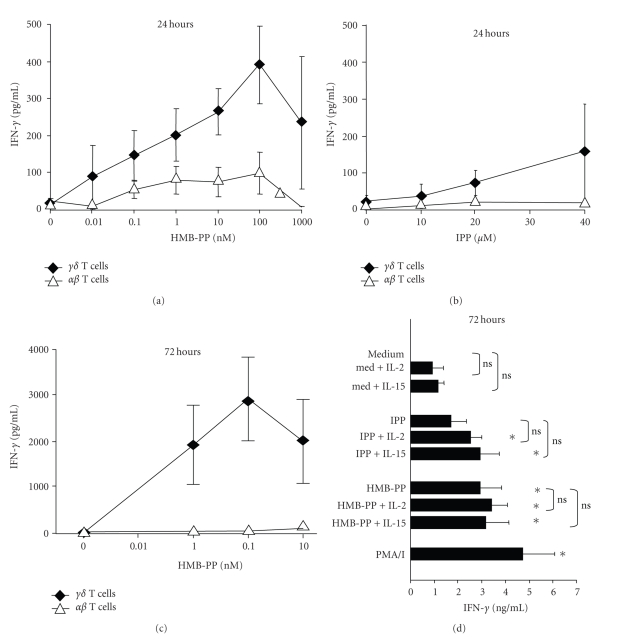
IFN-*γ* production in response to HMB-PP is restricted to *γδ* T cells and is highest 72 hours after simulation. Freshly isolated PBMCs were enriched for *αβ* and *γδ* T cells using magnetic beads, and 200,000 cells of each population were stimulated with various concentrations of HMB-PP (a and c) or IPP (b). The levels of IFN-*γ* in cell supernatants were quantified by ELISA after 24 hours (a and b) or 72 hours (c). Results are expressed as mean (± SEM) levels of IFN-*γ* released by *αβ* and *γδ* T cells from 2–8 donors. (d) Mean (± SEM) IFN-*γ* production by *γδ* T cells from 8 donors after stimulation with 10 *μ*M IPP, 10 nM HMB-PP, 50 U/mL IL-2, and/or 10 ng/mL IL-15 as indicated. *P < .05 compared to unstimulated cells (medium). ns: not significant comparing absence and presence of IL-2 and IL-15.

We also measured levels of IFN-*γ*, IL-10, IL-13, and IL-17 that were released into culture supernatants of pyrophosphate-stimulated *γδ* TCR^+^ and *γδ* TCR^−^ (denoted *αβ*) T cells using ELISA. *αβ* and *γδ* T cells were enriched using magnetic beads, and 0.2 × 10^6^ cells of each type were stimulated with various concentrations of HMB-PP or IPP in the absence or presence of IL-2 or IL-15. After 24 hours, IFN-*γ* was detectable in the supernatants of V*γ*9V*δ*2 T cells, with half-maximal levels seen when 20 *μ*M IPP or 1 nM HMB-PP were used (Figures 3(a) and 3(b)). In contrast, very low levels of IFN-*γ* were released by phosphoantigen-exposed *αβ* T cells. Measurement of IFN-*γ* levels 24, 48, and 72 hours after stimulation indicated that maximal amounts were detectable after 72 hours, with levels 5–10 times higher than observed after 24 hours (Figure 3(c)). 

Figure 3(d) shows that stimulation of V*γ*9V*δ*2 T cells with IPP or HMB-PP for 72 hours resulted in significant IFN-*γ* release. We found that after culturing unstimulated V*γ*9V*δ*2 T cells for 72 hours in the presence of IL-2 or IL-15, low levels (means 0.9 and 1.2 ng/mL, resp.) of IFN-*γ* were detectable in the culture supernatants. However, IL-2 or IL-15 only slightly augmented the amounts of IFN-*γ* released by phosphoantigen-stimulated V*γ*9V*δ*2 T cells in 3 experiments. Thus, although IL-2 and IL-15 increase the frequencies of pyrophosphate-stimulated V*γ*9V*δ*2 T cells that express IFN-*γ* at an early time point (Figure 2), they do not significantly enhance the overall levels of IFN-*γ* released by these cells after 72 hours. 

### 3.4. *γδ* T Cells Do Not Secrete IL-10, IL-13, Nor IL-17 in Response to Phosphoantigen Stimulation But Can Release IL-13 in Response to Treatment with IL-2 or IL-15

**Figure 4:**
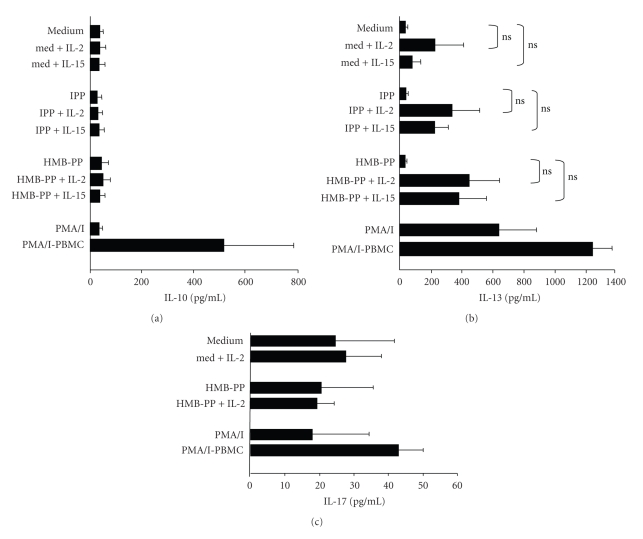
Phosphoantigen stimulation of *γδ* T cells causes no detectable increase in IL-10, IL-13, or IL-17 production. Freshly isolated PBMCs were enriched for *γδ* T cells using magnetic beads, and 200,000 cells were stimulated with 10 *μ*M IPP, 10 nM HMB-PP, 50 U/mL IL-2, and/or 10 ng/mL IL-15 as indicated. The levels of IL-10 (a), IL-13 (b), and IL-17 (c) in cell supernatants were measured by ELISA after 72 hours. Bar charts show mean cytokine levels for all treatments for 8 donors. ns: not significant comparing absence and presence of IL-2 and IL-15. PMA/I-PBMC, cytokine levels released by PMA and ionomycin-stimulated PBMC.

While enriched *γδ* T cells significantly upregulated IFN-*γ* in response to pyrophosphate stimulation (Figure 3), no upregulation of IL-10, IL-13, nor IL-17 was detected at any time point after stimulation (Figure 4). We observed that, in contrast to IFN-*γ*, even PMA and ionomycin treatment was insufficient to upregulate IL-10 nor IL-17 production by *γδ* T cells, even though these cytokines were released by PMA and ionomycin-treated total PBMC. Addition of IL-2 or IL-15 to unstimulated, IPP-stimulated, or HMB-PP-stimulated *γδ* T cells resulted in low level secretion of IL-13 (but not IL-10 nor IL-17) after 72 hours (Figure 4(b)). Therefore, while pyrophosphate stimulation did not induce IL-13 release by *γδ* T cells, some *γδ* T cells are nevertheless capable of producing this cytokine when cultured with the growth factors IL-2 or IL-15. 

### 3.5. Cytokine Production by V*γ*9V*δ*2 T Cells Is Independent of CD4 or CD8 Expression

**Figure 5:**
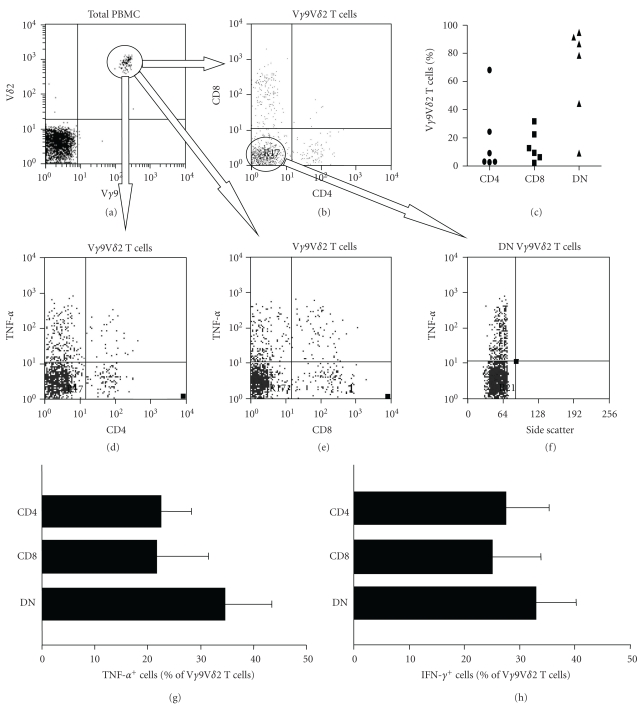
Cytokine expression by V*γ*9V*δ*2 cells is not influenced by CD4 or CD8 expression. PBMCs were stimulated with PMA and ionomycin and surface expression of V*γ*9, V*δ*2, CD4, and CD8, and intracellular expression of cytokines was analysed by flow cytometry after 4 hours. (a) V*γ*9^+^V*δ*2^+^ cells were gated initially and (b) CD4 and CD8 expression by gated V*γ*9V*δ*2 T cells was analysed. (c) Mean frequencies of CD4^+^, CD8^+^, and double-negative (DN) V*γ*9V*δ*2 T cells from 6 donors. (d, e) Flow cytometry dot plots showing expression of TNF-*α* by CD4^+^ and CD4^−^ (d) and CD8^+^ and CD8^−^ (e) V*δ*2V*γ*9 T cells stimulated with PMA and ionomycin. (f) Dot plot showing expression of TNF-*α* by gated DN V*γ*9V*δ*2 T cells. Arrows show electronic gates used to generate dot plots. (g, h) Frequencies of CD4^+^, CD8^+^, and DN V*δ*2V*γ*9 T cell that produced TNF-*α* (g) and IFN-*γ* (h) in response to stimulation with PMA and ionomycin. Results are means of 5 independent experiments.

Cytokine production by *αβ* T cells can vary according to whether they express CD4, CD8, or neither. We used flow cytometry to examine CD4 and CD8 expression by V*γ*9V*δ*2 T cells and found that >75% were negative for these coreceptors in 4 of 6 donors. However significant numbers of CD4^+^, CD8^+^, and double-negative V*γ*9V*δ*2 T cells were found in all subjects (Figures 5(a)–5(c)). We next investigated whether these subsets of V*γ*9V*δ*2 T cells can produce TNF-*α* or IFN-*γ*. Electronic gating on V*γ*9V*δ*2 T cells from 6 donors revealed that over 25% of CD4^+^ and CD8^+^ V*γ*9V*δ*2 T cells expressed TNF-*α* (Figures 5(d), 5(e), and 5(g)) and IFN-*γ* (Figure 5(h)) upon PMA and ionomycin stimulation. Electronic gating on CD4^−^CD8^−^ V*γ*9V*δ*2 T cells indicated that >35% of PMA and ionomycin-stimulated cells within this double-negative subset also produced these cytokines (Figures 5(f), 5(g), and 5(h)). Thus, cytokine production by V*γ*9V*δ*2 cells does not appear to correlate with CD4 or CD8 expression as for *αβ* T cells, with significant proportions of CD4^+^, CD8^+^, and double-negative V*γ*9V*δ*2 T cells capable of producing Th1 cytokines. 

## 4. Discussion

V*γ*9V*δ*2 T cells are innate lymphocytes that are thought to play key roles in immune sensing of danger and initiation and regulation of innate and adaptive immunity. They respond to a variety of signals including ligands for toll-like receptors [45, 46], stress-inducible ligands on infected and tumour cells [47], and phosphoantigens which may be produced endogenously or by microorganisms [1, 48]. They respond with a plethora of immunostimulatory functions, including cytotoxicity [5, 19–21], direct activation of other cells of the immune system [11–18], and indirect control of immune responses via the rapid secretion of chemokines and cytokines [3, 6, 7, 10, 11]. For these reasons, V*γ*9V*δ*2 T cells have attracted considerable interest as therapeutic agents for infectious and immune-mediated disease and cancer. Indeed, V*γ*9V*δ*2 T cells may perhaps be more rational candidates as immunotherapeutic targets than invariant natural killer T (iNKT) cells, which also recognise nonpeptide antigens via a conserved TCR and mediate antitumour immunity but are found at ~100-fold lower frequencies in human blood compared to V*γ*9V*δ*2 T cells [49].

Clinical trials using various V*γ*9V*δ*2 T cell activating agents, such as aminobisphosphonates and phosphoantigens, are ongoing for treatment of cancer [22–26]. Such trials report low toxicity and improved objective clinical outcomes including stabilisation and partial or full remission of advanced-stage metastatic carcinomas of the prostate, lung, and kidney. Therapeutic activation of V*γ*9V*δ*2 T cells may also prove to be beneficial in numerous other disease settings, due to their potent adjuvant and effector functions in innate and adaptive immunity [10–16]. However, it is conceivable that the same potent activities could lead to the activation of undesirable immune responses that could result in inflammatory or autoimmune disease; therefore, further characterisation of the mechanisms by which V*γ*9V*δ*2 T cells respond to different stimuli is required. 

In this study, we analysed the cytokine production profiles of V*γ*9V*δ*2 T cells treated *in vitro* in a similar manner to current immunotherapeutic protocols, that is, using phosphoantigens and T-cell growth factors. We used two phosphoantigens, the prototype IPP [48] and the more potent HMB-PP, which can induce proliferation, cytotoxicity, and cytokine secretion by V*γ*9V*δ*2 T cells even at subnanomolar concentrations [40, 41]. These antigens were used to stimulate freshly isolated V*γ*9V*δ*2 T cells *in vitro*, in the presence and absence of IL-2 and IL-15, and the early production of cytokines that help polarise adaptive immune responses was examined.

Our results show that V*γ*9V*δ*2 T cells promptly produce the proinflammatory cytokines IFN-*γ* and TNF-*α*, in response to phosphoantigen stimulation, with IFN-*γ*-producing cells detected as early as 4 hours after stimulation. In contrast, we were unable to detect any upregulation of IL-10, IL-13, or IL-17 production in response to phosphoantigen stimulation. This Th1 cytokine profile was observed exclusively in V*γ*9V*δ*2 T cells when freshly isolated PBMCs were stimulated with either IPP or HMB-PP, and intracellular cytokine expression was analysed by flow cytometry. The same profile was seen when magnetic bead-enriched *γδ* T cells were stimulated for up to 72 hours and released cytokine was quantified by ELISA. The frequencies of IFN-*γ* expressing V*γ*9V*δ*2 T cells and amounts of cytokine released followed dose-dependent responses to each pyrophosphate, with HMB-PP being ~10,000-fold more potent than IPP, in agreement with previous reports [41, 42]. 

The addition of IL-2 or IL-15 to pyrophosphate stimulations led to moderate increases in the proportions of V*γ*9V*δ*2 T cells that produced IFN-*γ* within 4 hours of stimulation, but these increases only translated into a slight enhancement in IFN-*γ* levels, when supernatants were analysed after 72 hours. This suggests that IL-2 and IL-15 speed up the kinetics of the V*γ*9V*δ*2 response, without synergising with phosphoantigens in inducing IFN-*γ* production, perhaps by stimulating cell division and proliferation of IFN-*γ*-expressing V*γ*9V*δ*2 T cells rather than directly stimulating IFN-*γ* secretion. This result may also be explained by the presence of other accessory cells, such as antigen-presenting cells [50], within the PBMC used for intracellular cytokine detection, which were absent from the enriched *γδ* T-cell preparations used for ELISA.

Interestingly, *γδ* T cells incubated with IL-2 or IL-15 in the absence of phosphoantigen stimulation released moderate amounts of IFN-*γ* and IL-13, but not IL-10 nor IL-17. This IFN-*γ* production was augmented by simultaneous stimulation with HMB-PP or IPP, but the IL-13 production was not. This suggests that phosphoantigen stimulation of V*γ*9V*δ*2 T cells results in secretion of IFN-*γ* but not IL-13, while treatment with IL-2 or IL-15 can induce secretion of both cytokines. Alternatively, the IFN-*γ* and IL-13 released in response to IL-2 or IL-15 may emanate from non-V*γ*9V*δ*2 T cells present in the enriched *γδ* T-cell preparations, such as V*δ*1^+^ T cells. However, our results clearly show that V*γ*9V*δ*2 T cells release IFN-*γ* and not IL-13 in response to phosphoantigen stimulation. 

Following confirmation that phosphoantigen-stimulated V*γ*9V*δ*2 T cells produce IFN-*γ* and TNF-*α*, we next investigated whether this cytokine profile is restricted to CD4^+^, CD8^+^, or double-negative V*γ*9V*δ*2 T cells. We found that all 3 subtypes of V*γ*9V*δ*2 T cells were found in all 6 donors tested, with double negative cells predominating. Significant proportions of all 3 V*γ*9V*δ*2 T cell subsets produced IFN-*γ* and TNF-*α* upon stimulation, showing CD4 or CD8 expression does not impact on cytokine production by these cells. 

The present findings are in agreement with previous studies that have shown that phosphoantigen-stimulated V*γ*9V*δ*2 T cells release cytokines that promote Th1 responses [12, 14, 33–37]. We also confirm that IL-2 or IL-15 augment Th1 cytokine production by these cells [36, 38]. We show, for the first time, that the CD4^+^, CD8^+^, and double-negative V*γ*9V*δ*2 T cell subsets all exhibit this Th1 cytokine profile. Our data fit with previous observations from us and others that phosphoantigen-stimulated V*γ*9V*δ*2 T cells possess Th1-promoting adjuvant activity for dendritic cells, inducing them to mature into antigen-presenting cells that release IL-12 but not IL-10 [14, 16], although Eberl and coworkers [12] reported that V*γ*9V*δ*2 T cells can induce maturation of monocytes into antigen-presenting cells that stimulated Th1 and Th17 cells. Although V*γ*9V*δ*2 T cells appear to be Th1 inducers, other studies have shown that subsets of these cells can release other Th cell-polarising cytokines under different conditions. Wesch and co-workers [35] reported that V*γ*9V*δ*2 T cells stimulated with IPP in the presence of IL-12 and anti-IL-4 mAb (Th1-priming conditions) produced IFN-*γ* and TNF-*α*, but when stimulated in the presence of IL-4 and anti-12 mAb (Th2 priming) they produced IL-4. Vermijlen and co-workers [38] reported that V*γ*9V*δ*2 T cells stimulated with HMB-PP and IL-2 can also produce IL-5 and IL-13, but in agreement with our results, these authors also found that treatment of V*γ*9V*δ*2 T cells with IL-2 in the absence of phosphoantigen stimulation was sufficient to induce IL-13 production [38]. A minor subset of V*γ*9V*δ*2 T cells characterised by the expression of CXCR5 and CD27 has also been reported to release IL-4 and IL-10 following stimulation with a synthetic phosphoantigen [11]. IL-17 and IL-22 production by a minority (<0.1%) of V*γ*9V*δ*2 T cells stimulated with HMB-PP in the presence of IL-1*β*, TGF-*β* and IL-6, or IL-23 has also been reported [39]. Thus, it is clear that some V*γ*9V*δ*2 T cells can be induced to produce various cytokines, but whether they do so under physiological conditions is unclear. Furthermore, such cells appear to account for very small proportions of peripheral blood V*γ*9V*δ*2 T cells, which under the conditions used in the present study make very small contributions to the overall cytokine profile of phosphoantigen-stimulated V*γ*9V*δ*2 T cells.

In conclusion, we have shown here that IPP and the novel phosphoantigen HMB-PP rapidly activates a strong proinflammatory response specific to V*γ*9V*δ*2 T cells and thus shows promise as an immunotherapeutic agent, worthy of further evaluation. Furthermore, we confirm that V*γ*9V*δ*2 T cells are also responsive to lymphocyte growth factors. These responses seen with fresh, uncultured V*γ*9V*δ*2 T cells are similar to those seen with V*γ*9V*δ*2 T cells that were expanded *in vitro* with HMB-PP and IL-2 [16]. Future studies are required to ascertain the effects of stimulating V*γ*9V*δ*2 T cells through other non-TCR stimulatory receptors, such as toll-like receptors [45, 46] and NKG2D [47] and inhibitory receptors [51], and the effects of ligating various costimulatory and inhibitory receptors. Clarification of these responses may allow selective induction of particular V*γ*9V*δ*2 T-cell subsets or functions and thus greatly enhance the gamut of immunotherapeutic tools available.
